# Mendelian randomization implicates blood lipids as an actionable mediator of the effect of basal metabolic rate on Alzheimer's disease

**DOI:** 10.1002/alz70861_108458

**Published:** 2025-12-23

**Authors:** Yunyun Guo

**Affiliations:** ^1^ The Ageing Epidemiology (AGE) Research Unit, School of Public Health, Imperial College London, London UK

## Abstract

**Background:**

Alzheimer's disease (AD) is a serious global health burden that disproportionately affects populations with low basal metabolic rate (BMR). This study aimed to obtain causal estimates of the association between BMR and AD and to quantify the mediating effects of known modifiable risk factors.

**Method:**

This study employed a two‐step, two‐sample multivariate Mendelian randomisation (MR) technique using SNPs as genetic tools for exposure (BMR) and mediator (lipid levels), thus minimising bias due to confounders and reverse causality. MR analyses were dominated by inverse variance weighting (IVW) and supplemented by a series of sensitivity analyses to verify the robustness of the results. We utilised pooled data from genome‐wide association studies of BMR, the proposed mediators (i.e. LDL cholesterol, HDL cholesterol, total cholesterol, triglyceride) and AD. The overall effect of BMR on AD was decomposed into direct effects and indirect effects via multiple mediators.

**Result:**

BMR was negatively associated with AD (OR = 0.78, 95% CI: 0.72 to 0.92, *p* = 0.001, IVW). The protective effect of high BMR on AD persisted even after considering anthropometric measures separately. Individually, the largest contributor was total cholesterol (38.65% mediation; 95%CI 15.34%, 61.97%), followed by triglyceride (23.51% mediation; 95%CI 0.08%, 46.94%) and LDL cholesterol (14.53% mediation; 95% CI 3.49%, 25.58%).

**Conclusion:**

These results support a potential causal protective effect of BMR on AD, mediated by a substantial proportion of modifiable lipid risk factors. Thus, interventions targeting these factors have the potential to significantly reduce the burden of AD due to low BMR.

